# Exploring in vivo cholesterol-mediated interactions between activated EGF receptors in plasma membrane with single-molecule optical tracking

**DOI:** 10.1186/s13628-016-0030-5

**Published:** 2016-06-24

**Authors:** Chien Y. Lin, Jung Y. Huang, Leu-Wei Lo

**Affiliations:** Department of Photonics, Chiao Tung University, 1001 Ta-Hsueh Road, Hsinchu, Taiwan; The T.K.P. Research Center for Photonics, Chiao Tung University, 1001 Ta-Hsueh Road, Hsinchu, Taiwan; Institute of Biomedical Engineering and Nanomedicine, National Health Research Institutes, 35, Keyan Road, Zhunan, Taiwan

**Keywords:** Epidermal growth factor receptor, Diffusion, Single-molecule trajectory, Live cell, Plasma membrane

## Abstract

**Background:**

The first step in many cellular signaling processes occurs at various types of receptors in the plasma membrane. Membrane cholesterol can alter these signaling pathways of living cells. However, the process in which the interaction of activated receptors is modulated by cholesterol remains unclear.

**Methods:**

In this study, we measured single-molecule optical trajectories of epidermal growth factor receptors moving in the plasma membranes of two cancerous cell lines and one normal endothelial cell line. A stochastic model was developed and applied to identify critical information from single-molecule trajectories.

**Results:**

We discovered that unliganded epidermal growth factor receptors may reside nearby cholesterol-riched regions of the plasma membrane and can move into these lipid domains when subjected to ligand binding. The amount of membrane cholesterol considerably affects the stability of correlated motion of activated epidermal growth factor receptors.

**Conclusions:**

Our results provide single-molecule evidence of membrane cholesterol in regulating signaling receptors. Because the three cell lines used for this study are quite diverse, our results may be useful to shed light on the mechanism of cholesterol-mediated interaction between activated receptors in live cells.

## Background

Receptor proteins are ubiquitous in the plasma membrane of mammalian cells, which transduce information about cellular environment to intracellular signaling networks [1–3]. There are approximately 1352 receptor proteins coded by human genome [4]. The flow of information through those receptors is critically shaped by receptor interactions. The diverse cellular processes regulated by such receptor proteins include cell growth and division, differentiation, migration, and apoptosis [5]. Receptor signaling dysregulation is attributed to the pathogenesis of several diseases [6, 7]. Therefore, understanding the interactions, molecular processes and relevant structures of such signaling receptors is imperative.

Human receptor proteins, including the epidermal growth factor receptor (EGFR), share a common molecular architecture, consisting of an extracellular binding domain, a single transmembrane helix, a flexible juxtamembrane segment, and an intracellular tyrosine kinase domain [8]. Ligand binding to the extracellular domains induces conformational reorganization that promotes receptor dimerization, leading to the activation of the intracellular tyrosine kinase domain. However, conclusions about the mechanism of ligand-induced dynamic recruitment and dimerization of membrane-associated receptors remains controversial [9]. Conventional steady-state ensemble approaches cannot be used to address the stochastic nature of activated receptors that encounter each other in a highly heterogeneous and fluidic plasma membrane. Recent advancements in single-molecule fluorescent imaging and tracking have provided further insights into the behavior of EGFR in vivo [10–12]. For example, Lidke et al. devised a two-color quantum-dot tracking method to visualize the state-dependent dimerization processes of human EGFR in living cells [13]. A hidden Markov model was used to extract the kinetic parameters of the underlying free, co-confined, and dimerized states.

Researchers have increasingly determined that lipid domains rich in glycosphingolipids and cholesterol can facilitate signaling receptors to form a dimer [13–15]. Because the recruitment mechanism and dynamic clustering of receptors in the hierarchical structure of plasma membranes are not clearly understood, the existence and functionality of lipid nanoscale domains on EGFR dimerization remains controversial. A growing body of research indicates that membrane cholesterol can influence the organization, stability, and function of membrane proteins including receptors [16–18]. The process by which this cholesterol modulates membrane proteins remains unclear.

The plasma membranes of live cells are complex and highly heterogeneous. Single-molecule tracking had been successfully used to probe the microscopic environments and fluctuations faced by receptor proteins in a living cell [13, 19, 20]. As a receptor protein diffuses in the plasma membrane, it encounters two types of interactions with its local environment [9, 21]. Firstly, the receptor protein can induce a local ordering of the surrounding lipid molecules via a lipid–protein interaction. Furthermore, confinement by actin filaments can also be involved. We developed an energetic model to describe the dynamic diffusion of a membrane-associated protein in a hierarchical structure of actin corrals and lipid domains [22]. This model enables us to identify critical information from single-molecule trajectories. Specifically, the confinement influences on EGFRs can be revealed by analyzing the normalized variance of mean square of diffusion step size of receptor proteins in live HeLa cells [20, 22]. In ref. [20], we focused on the influence of lipid domains on the diffusion of EGFRs. However, affinity of liganded EGFR for lipid domains and requirement of membrane cholesterol have not been verified on different cell lines. In this study, we employed single-molecule tracking technique to address the problem associated with the effect of membrane cholesterol on receptor-receptor interaction in vivo. Please note that those data are spatially and temporally coarse-grained from sampling of real molecule motions. The diffusion coefficient deduced from the coarse-grained trajectories can be location-dependent, reflecting the influences from local barriers [23].

Three cell lines were carefully selected for this study. HeLa cells, which was derived from cervical cancer cells and had served as the standard in cellular biology research. Typically, HeLa cells can express EGFR at a level of 20,000 per cell [24] and approximately contain 17 *μ*g cholesterol per mg protein [25]. The second cell line, A431, derived from a human epidermal carcinoma, is a model system for the study of cancer-associated cellular signaling pathways. A431 cells can express EGFR at an extremely high level (500,000 per cell) [24] and contain 32 *μ*g cholesterol per mg protein [26]. The third one, MCF12A, is a non-tumorigenic breast epithelial cell line, which expresses EGFR normally.

## Methods

**Fig. 1:**
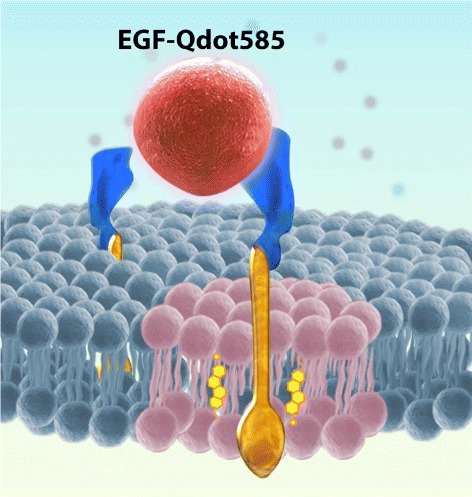
Schematic diagram of epidermal growth factor receptors (EGFRs) in the environment of plasma membrane. EGF-Qdot585 is a fluorescent epidermal growth factor (EGF) synthesized by conjugating EGF with a quantum dot Qdot585. Here cholesterol molecules are shown in *yellow*, the *pink* lipids represent the raft lipid species, and the *dark green* lipids are nonraft lipids

EGFRs were labeled to display their diffusive motions in the plasma membranes of live cells. An anti-EGFR antibody-quantum dot complex was synthesized for attaching specifically on unliganded EGFRs as a fluorescent tag. To label liganded EGFRs, we used fluorescent epidermal growth factors (EGF) to activate EGFRs and tag them at the same time. A schematic diagram of native and liganded EGFRs in the environment of plasma membrane is described in Fig. 1.

### Cell culture and reagents

HeLa and A431 cells were cultured in Dulbecco’s Modified Eagle’s medium (DMEM) with 10 *%* (v/v) fetal bovine serum without phenol red, whereas MCF12A cells were cultured in a 1:1 mixture of DMEM and Ham’s F12 medium containing 20 ng/mL Human EGF, 0.01 mg/mL bovine insulin, 500 ng/mL hydrocortisone, and 5 *%*(*v*/*v*) horse serum. Before the single-molecule live-cell imaging was performed, the cells were plated in a slide with eight-well chambers. After a 70–80 *%* confluence was reached, HeLa and A431 cells were deprived of serum for 24 h, whereas MCF12A cells were deprived of serum for 3 h.

To tag EGFRs in the plasma membranes of live cells, anti-EGFR antibody (Thermo Fisher Scientific, Waltham, MA, USA) was first biotinylated. The resulting biotinylated anti-EGFRs were conjugated with quantum dots (Qdot585-streptavidin, Thermo Fisher Scientific). Cells were incubated in a cell culture containing 10 nM of anti-EGFR-Qdot585 for 15 min and then washed three times with phosphate buffered saline (PBS). Fluorescent EGF (EGF-Qdot585) was synthesized by conjugating biotin-EGF from Invitrogen to Qdot585-streptavidin in PBS. To activate EGFRs, cells were incubated in a cell culture containing 40 ng/mL of EGF-Qdot585. Fluorescent anti-CD59 antibody was synthesized by biotinylating anti-CD59 antibody (Sigma-Aldrich, St. Louis, MO, USA) and then conjugated with Qdot585-streptavidin in PBS. To label CD59, we followed the same protocol as described above for labeling EGFR.

In this research, we used two drugs to modify the amount and spatial distribution of cholesterol in the plasma membranes of live cells. The first drug is nystatin, which can be used to render cholesterol more uniformly distributed, while keeping the total amount of cholesterol constant [27]. The second drug is methyl- *β*-cyclodextrin (M *β*CD), which can deplete membrane cholesterol to a low level [28]. Cells were incubated in their normal growth medium containing 7.5 mM M *β*CD for 1 h and then washed three times with PBS before EGFRs (or CD59) in the cells were labeled fluorescently. To create a uniform cholesterol distribution, cells were incubated in a cell culture with 10 *μ*g/mL nystatin for 1 h.

### Single-molecule optical measurement

The output from a blue (473 nm) solid-state laser was used to excite quantum dots in live cells. The fluorescent signals were collected with a high numerical aperture (NA) oil immersion objective lens mounted on an inverted optical microscope (IX-71, Olympus Optical Co., Tokyo, Japan) and filtered using a 473-nm Raman notch filter. We then detected the fluorescent signals with an electron-multiplying charge-coupled device (EMCCD, Cascade II 512, Photometrics Inc., Huntington Beach, CA, USA).

### Data analysis

**Fig. 2:**
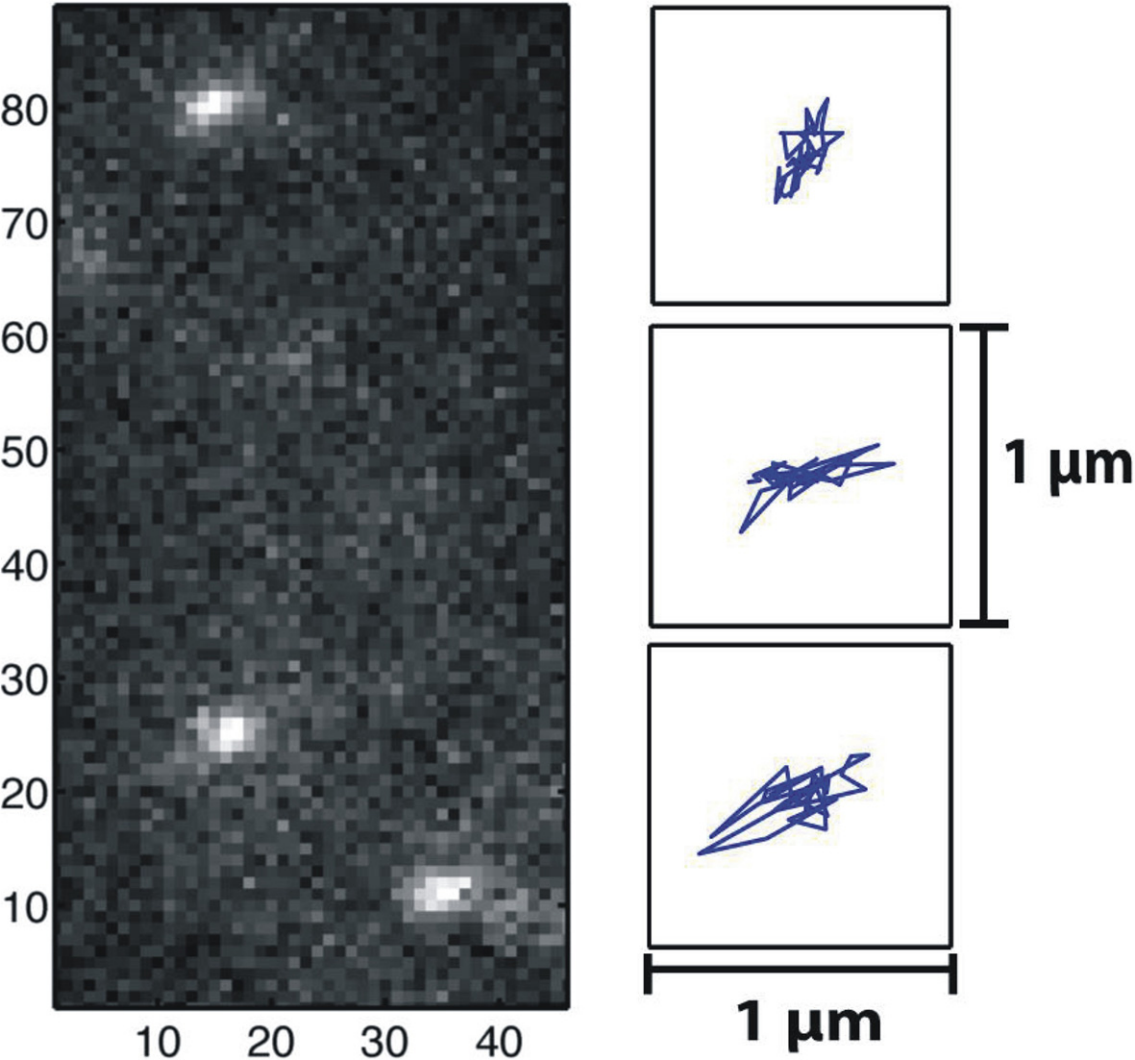
An EMCCD image of single-molecule EGFRs in a living cell taken with an exposure time of 25 ms; Three typical trajectories of EGFRs diffusing in a confined region were extracted from measured trajectories using the confinement quantification procedure [30]

Single-molecule optical trajectories of proteins under study were recorded for as long as 100 s with a frame period of *τ*=25 ms. Position coordinates of single-molecule proteins were extracted from a set of images. A typical EMCCD image of EGFRs in a living cell is presented in Fig. 2. The nearest positions in consecutive frames were connected to form a single-molecule trajectory by using multiple-target tracing algorithm [29]. Events of confined diffusion were extracted from a single-molecule trajectory by using the confinement quantification procedure [30]. The mean squared displacements (MSD) $\overline {{{R}_{\tau }}^{2}(t)}=\overline {\left [ |\vec {r}(t+\tau)-\vec {r}(t){{|}^{2}}+|\vec {r}(t)-\vec {r}(t-\tau){{|}^{2}} \right ]/2}$ were calculated from single-molecule trajectories. The localization accuracy of our apparatus was approximately 40 nm, implying an accuracy of 0.002 *μm*^2^ for $\overline {{{R}_{\tau }}^{2}}$ determination. We presented a histogram of MSD and normalized variance $V(\overline {{{R}_{\tau }}^{2}})={{\sigma }^{2}}({{R}_{\tau }}^{2})/\overline {{{R}_{\tau }}^{2}}$ in a contour plot; here $\overline {{{R}_{\tau }}^{2}(t)}$ can quantify the manner in which a receptor molecule diffuses in its environment, and $V(\overline {{{R}_{\tau }}^{2}})$ can reveal the nature (deterministic or stochastic) of interaction between a receptor protein and its environment [22]. An attractive feature of this plot is that when a molecule repeatedly visits a membrane domain, the characteristic $V(\overline {{{R}_{\tau }}^{2}})$ and $\overline {{{R}_{\tau }}^{2}}$ of the lipid domain is imposed on the trajectories, resulting in the formation of a peak at the corresponding position on the plot.

The mathematical foundation of the method had been detailed in [22]. Here we summarize some key findings to facilitate further discussion. For molecules under free diffusion, $V(\overline {{{R}_{\tau }}^{2}})$ has a constant value of 2. As a receptor protein diffuses under a strong confinement of actin corral, $V(\overline {{{R}_{\tau }}^{2}})$ can be a large positive value [20]. By contrast, $V(\overline {{{R}_{\tau }}^{2}})$ of a receptor is reduced to below 2 when it is confined in a lipid domain. This can be understood as follows: Actin filaments act as soft potential barriers for the diffusing protein [21, 22, 31]. As membrane proteins diffuse near an actin filament, the soft potential barrier may stall the diffusing proteins for a brief moment, which causes a large variance in the diffusion step size. Furthermore, the faster the protein diffuses, the larger the variance is. At the limit of fast diffusion, $V(\overline {{{R}_{\tau }}^{2}})$ can reach a saturated level, depending on the barrier height. Owing to the protein-lipid interaction, we can view a protein and its nearby ordered lipids as a dressed protein. For a protein diffusing in a lipid domain, the faster the protein diffuses, the larger the dressing effect is. This shall result in a smaller variance of $\overline {{{R}_{\tau }}^{2}}$, and thereby yields smaller $V(\overline {{{R}_{\tau }}^{2}})$ for faster diffusing proteins.

## Results

### Single-molecule optical trajectories of CD59 reveal the association with cholesterol-rich lipid domains

Lipid raft domains are rich in cholesterol and glycosphingolipids such as ganglioside GM1 [15]. Previous studies had reported that CD59 can colocalizes with GM1 lipids in the plasma membrane of a cell [32, 33]. Here we used CD59 as a marker for the lipid raft domain.

**Fig. 3:**
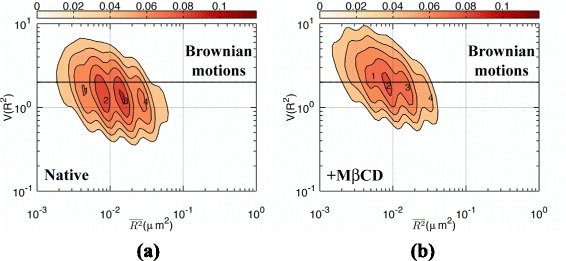
Two-dimensional contour plot on the $V(\overline {{{R}_{\tau }}^{2}})$-$\overline {{{R}_{\tau }}^{2}}$ plane for Qdot585-Ab-CD59 diffusing in the plasma membrane of (**a**) native HeLa cells and (**b**) M *β*CD-pretreated HeLa cells

We analyzed the trajectories of Qdot585-Ab-CD59 in HeLa cells. The resulting $V(\overline {{{R}_{\tau }}^{2}})$-$\overline {{{R}_{\tau }}^{2}}$ contour plot was presented in Fig. 3. For the native HeLa cells, Fig. 3a displays four peaks (labeled 1, 2, 3, and 4) at the $(\overline {{{R}_{\tau }}^{2}}, V(\overline {{{R}_{\tau }}^{2}}))$ coordinates of (0.004, 1.6), (0.008, 1.2), (0.015, 1.2), and (0.028, 1.2), respectively. For the M *β*CD-treated cells (see Fig. 3b), the four peaks shift upwards as $V(\overline {{{R}_{\tau }}^{2}})$ approaches the free diffusion limit. This result is consistent with the assumption that CD59 proteins are embedded in lipid raft domains. When membrane cholesterol is depleted by M *β*CD, CD59s are released from lipid raft domains, allowing them to diffuse freely.

### EGF binding causes EGFRs to translocate into cholesterol-rich lipid domains.

**Fig. 4:**
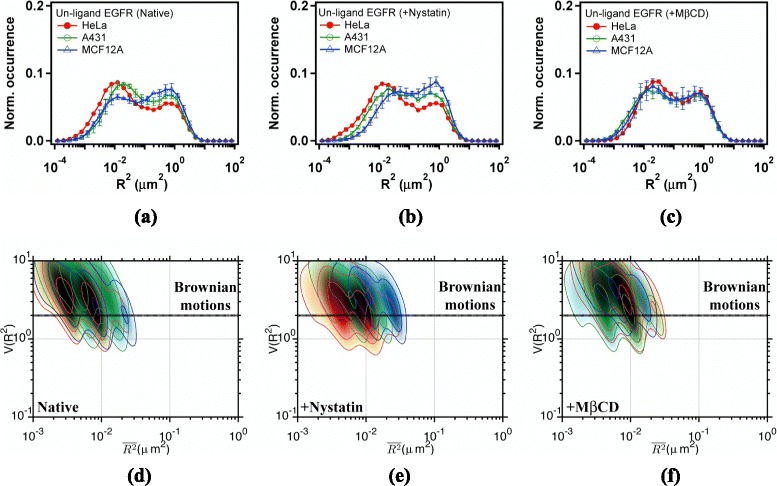
**a**, **b**, **c** Histograms of mean-square displacements (MSD) and (**d**, **e**, **f**) $V(\overline {{{R}_{\tau }}^{2}})$-$\overline {{{R}_{\tau }}^{2}}$ plots of unliganded EGFR (Qdot585-Ab-EGFR) diffusing in the plasma membrane of (**a**, **d**) native cells, (**b**, **e**) nystatin-pretreated cells, and (**c**, **f**) M *β*CD-pretreated cells. Trajectories were sampled with a frame period of *τ*=25 ms. Data are shown in *red* for HeLa cells, *green* for A431 cells, and *blue* for MCF12A cells

Equipped with the result of CD59, we moved on to measure and analyze the trajectories of unliganded EGFRs in the three cell lines. By grouping the MSDs to an appropriate number of bins, a histogram of MSD can be prepared (see Fig. 4a). Note that the diffusion coefficient is related to MSD by $D={R_{\tau }^{2}(t)}/{4 \tau }$ with a frame period of *τ*=25 ms. We can identify two groups of diffusing species in the histogram [20]. The faster group displays a peak at about 0.8 *μm*^2^, attributable to non-confined diffusion. The slower group has a peak near 0.012 *μm*^2^, which can be ascribed to confined diffusion. Unliganded EGFRs of the two cancerous cell lines (HeLa and A431) prefer to diffuse in the confined diffusion state. The reverse is apparent in the normal epithelial MCF-12A cells. The corresponding $V(\overline {{{R}_{\tau }}^{2}})$-$\overline {{{R}_{\tau }}^{2}}$ plots of unliganded EGFRs in the confined diffusion state are illustrated in Fig. 4d for HeLa (Red), A431 (Green), and MCF-12A (Blue), respectively. The regions colored in black indicate the similarity of the confined diffusion state in the three cell lines. Two peaks with $V(\overline {{{R}_{\tau }}^{2}})$ values of 3 and 4 occur in the three cell lines, indicating a strong interaction of EGFR with actin corrals [22].

When different drug effects on membrane cholesterol are applied with nystatin and M *β*CD, cholesterol-mediated interaction of EGFRs can be investigated. The results are shown in Fig. 4b and c. For the nystatin-pretreated cells, the MSD profile of EGFR is similar to that in native cells. Remarkably in native MCF-12A cells, the population of unliganded EGFR diffusing in the fast state is larger, whereas that in the M *β*CD-treated cells it becomes smaller. After M *β*CD treatment the cell line dependent variations in MSD profiles become smaller (see Fig. 4c), revealing that the lipid environments of unliganded EGFRs in the three cell lines are similar after membrane cholesterol is depleted. All these observations clearly indicate that the diffusion of unliganded EGFR is relevant to the amount of membrane cholesterol. Figure 4e and f display the corresponding $V(\overline {{{R}_{\tau }}^{2}})$-$\overline {{{R}_{\tau }}^{2}}$ plots of unliganded EGFR in the cell lines subjected to different drug treatments. Similar $V(\overline {{{R}_{\tau }}^{2}})$-$\overline {{{R}_{\tau }}^{2}}$ values as those observed in native cells were observed, which is opposite to that of CD59 presented in Fig. 3. The result suggests that unliganded EGFR may locate outside of the cholesterol-rich lipid domains.

**Fig. 5:**
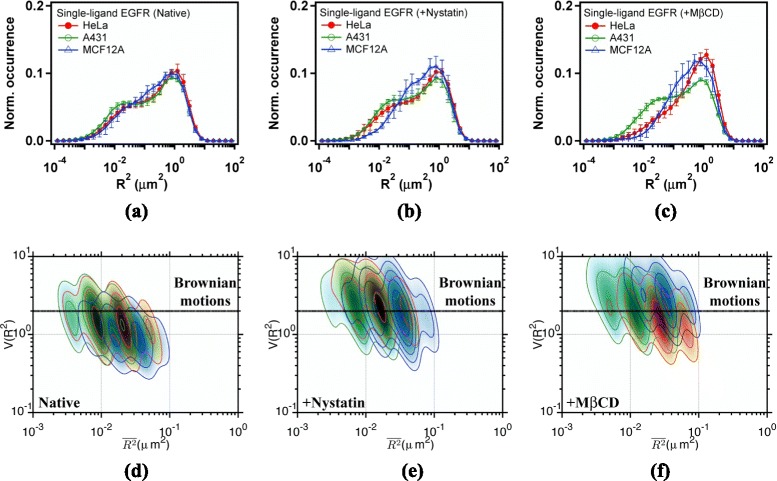
**a**, **b**, **c** Histograms of MSDs and (**d**, **e**, **f**) $V(\overline {{{R}_{\tau }}^{2}})$-$\overline {{{R}_{\tau }}^{2}}$ plots of liganded EGFR (Qdot585-EGF-EGFR) diffusing in the plasma membrane of (**a**, **d**) native cells, (**b**, **e**) nystatin-pretreated cells, and (**c**, **f**) M *β*CD-pretreated cells. Trajectories were sampled with a frame period of *τ*=25 ms. Data are shown in *red* for HeLa cells, *green* for A431 cells, and *blue* for MCF12A cells

Figure 5a depicts the diffusion behavior of liganded EGFR in the three cell lines. Comparing to the result of unliganded EGFR shown in Fig. 4a, we found that receptor activation with EGF appears to promote the liganded EGFR (Qdot585-EGF-EGFR) to diffuse in the fast state. Figure 5b and c show the effects of nystatin and M *β*CD on liganded EGFR. Among the three cell lines, the diffusion behavior of liganded EGFRs in A431 cells is least sensitive to changes in membrane cholesterol. The MSD profile of liganded EGFR in native HeLa cells is similar to that in A431; however, it becomes more similar to that in MCF-12A after M *β*CD treatment (Fig. 5c).

Figure 5d presents the corresponding $V(\overline {{{R}_{\tau }}^{2}})$-$\overline {{{R}_{\tau }}^{2}}$ plots of native cells. Compared to the result of unliganded EGFR shown in Fig. 4d, EGF binding decreases $V(\overline {{{R}_{\tau }}^{2}})$ to below the free diffusion limit, suggesting that liganded EGFR may encounter with different environments. The $V(\overline {{{R}_{\tau }}^{2}})$ value of liganded EGFR increases from that in the native cells (Fig. 5d) to approximately at the free diffusion limit in the nystatin-pretreated (Fig. 5e) and M *β*CD-pretreated cells (Fig. 5f). By contrast, uniformly distributing membrane cholesterol does not change the $V(\overline {{{R}_{\tau }}^{2}})$ value of unliganded EGFR. This finding suggests that liganded EGFRs may colocalize with membrane cholesterol, but unliganded EGFRs do not. The $V(\overline {{{R}_{\tau }}^{2}})$-$\overline {{{R}_{\tau }}^{2}}$ plots of liganded EGFRs in the three cell lines are similar to that of CD59, further supporting that these two types of proteins are located in similar environments. These experimental results may be effectively explained by the concept that unliganded EGFRs are located outside the cholesterol-rich lipid domains and EGF binding causes the receptors to move into the cholesterol-rich lipid domains [20].

### Cholesterol-mediated interaction between liganded EGF Receptors

When a receptor protein passes a nearby receptor, it may experience an attractive force that can result in a correlated motion between the two molecules [34]. To quantitatively display the correlation between two trajectories, we expressed the position vectors as a phasor ${\vec r_{k}}(t) = {A_{k}}(t){e^{i\,{\theta _{k}}(t)}}$ and calculated the degree of correlation with

1 $$ \begin{aligned} C(\tau) &= \text{Re} \left[ {{{\sum {{{\vec r}_{1}}^{*}(t) \cdot {{\vec r}_{2}}(t + \tau)}} \over {\sqrt {{{\sum {\left| {{{\vec r}_{1}}(t)} \right|} }^{2}}} \sqrt {{{\sum {\left| {{{\vec r}_{2}}(t)} \right|} }^{2}}} }}} \right]\\ &= {{{\sum\nolimits}_{t} {{A_{1}}(t){A_{2}}(t + \tau)\cos \left[{\theta_{2}}(t + \tau) - {\theta_{1}}(t)\right]}} \over {\sqrt {{\sum\nolimits}_{t} {{A_{1}}^{2}}} \sqrt {{\sum\nolimits}_{t} {{A_{2}}^{2}}} }}. \end{aligned}   $$

Thus, we can select highly correlated segments from trajectories by using *C*>0.8. We analyzed those segments to disclose the correlated motion of paired Qdot585-EGF-EGFRs.

**Fig. 6:**
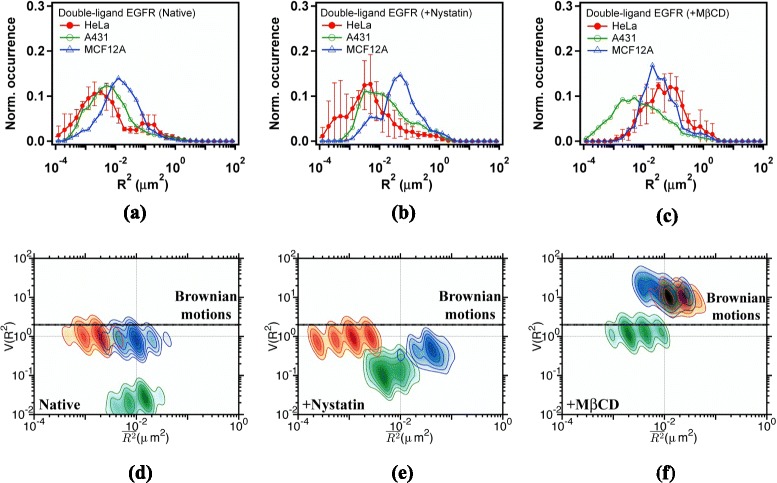
**a**, **b**, **c** Histograms of MSDs and (**d**, **e**, **f**) $V(\overline {{{R}_{\tau }}^{2}})$-$\overline {{{R}_{\tau }}^{2}}$ plots of correlated Qdot585-EGF-EGFRs diffusing in the plasma membrane of (**a**, **d**) native cells, (**b**, **e**) nystatin-pretreated cells, and (**c**, **f**) M *β*CD-pretreated cells. Trajectory segments with a degree of correlation exceeding 0.8 were analyzed. Trajectories were sampled with a frame period of *τ*=25 ms. Data are shown in *red* for HeLa cells, *green* for A431 cells, and *blue* for MCF12A cells

Figure 6a displays the MSD histograms of correlated Qdot585-EGF-EGFRs in native HeLa (red), A431(green), and MCF-12A (blue), respectively. The correlated Qdot585-EGF-EGFRs in HeLa and A431 cells appear to diffuse more slowly than the independent Qdot585-EGF-EGFR does, reflecting an attractive interaction effect with their nearby companion. To further examine the nature of receptor-receptor and receptor-environment interactions, we subjected those highly correlated segments to $V(\overline {{{R}_{\tau }}^{2}})$-$\overline {{{R}_{\tau }}^{2}}$ analysis. The resulting $V(\overline {{{R}_{\tau }}^{2}})$-$\overline {{{R}_{\tau }}^{2}}$ plots for the three cell lines are presented in Fig. 6d. The contour plots are more scattered than those of independent Qdot585-EGF-EGFRs (see Fig. 5d), revealing that this data analysis scheme is highly sensitive to receptor interaction. The $V(\overline {{{R}_{\tau }}^{2}})$ value of the correlated Qdot585-EGF-EGFRs is considerably lower in A431 cells, attributable to highly effective receptor-lipid and receptor-receptor interactions in A431 cells [20]. To inspect the nature of interactions and the relevance to receptor-induced lipid ordering, we again took advantages of the drug effects with nystatin and M *β*CD. Figure 6b and c display the MSD histograms of the correlated Qdot585-EGF-EGFRs. Pretreatment of HeLa cells with M *β*CD shifts the MSD profile of liganded EGFRs to the side of higher diffusion constant. M *β*CD treatment also broadens the MSD profile of liganded EGFRs in A431 cells. The $V(\overline {{{R}_{\tau }}^{2}})$-$\overline {{{R}_{\tau }}^{2}}$ plots for the three cell lines pretreated with nystatin are presented in Fig. 6(e). Correlated Qdot585-EGF-EGFRs appear to experience a weaker interaction in the nystatin-treated A431 cells, as evidenced by an increased $V(\overline {{{R}_{\tau }}^{2}})$ value. This observation may be explained with less stable lipid domain due to lower amount of cholesterol, resulting in larger variance of diffusing step size of the correlated receptors. By contrast, the effective interaction becomes stronger in nystatin-treated MCF-12A cells, suggesting the effect of cholesterol-mediated interaction is opposite to that of receptor-lipid interaction. The $V(\overline {{{R}_{\tau }}^{2}})$ of correlated Qdot585-EGF-EGFR in A431 increases by two orders of magnitude from 10^−2^ of native cells to 1 of M *β*CD treated cells. Noteworthy, the $V(\overline {{{R}_{\tau }}^{2}})$ value can be increased to higher than 10 in M *β*CD treated HeLa and MCF-12A cells, revealing that a deterministic dimerization interaction will dominate after membrane cholesterol is depleted. These observation results exhibit the vital role of membrane cholesterol in mediating the interaction between liganded receptors in the three cell lines under study.

## Discussion

A receptor protein can induce order in the surrounding lipids through the receptor-lipid interaction [22]. The degree of induced lipid order is determined by the effects of receptor-lipid interaction and the amount of cholesterol in the plasma membrane. Recent molecular dynamics (MD) simulations of human receptor tyrosine kinases in various lipid bilayers revealed that the predominant drivers of the receptor-induced lipid ordering domains may originate from electrostatic interactions between the anionic lipids and clustering basic residues in the juxtamembrane starting region of receptors [35]. Lipid domains had been predicted to exist in multicomponent membranes and experimentally observed on artificial membranes. For example, thermal fluctuations on a multicomponent membrane can produce inhomogeneities of lipid phases because the order parameters of lipid systems depend not only on the lipid composition but also on the compositional difference of two lipid leaves [36]. Coupling between inner and outer leaves of an asymmetric lipid bilayer could also produce inhomogeneities of lipid phases with a nonzero curvature [37].

In a cholesterol-rich lipid domain, interactions between receptors may be regulated by membrane cholesterol. Such cholesterol-mediated interactions between membrane-associated proteins and cholesterol-dependent nanoassemblies had been reported in the literature [16, 38–40]. Recently, coarse-grained MD simulations had been carried out to explore the nature of molecular interaction between membrane cholesterol and *β*_2_-adrenergic receptor [41]. This simulation revealed that cholesterol can bind to transmembrane helix IV of *β*_2_-adrenergic receptor and thereby regulates the dimer formation. Several experimental studies also showed that membrane cholesterol can regulate ligand-induced activation of receptors [16–18]. Affinity of liganded EGFR for lipid domains and requirement of membrane cholesterol in those lipid heterogeneities have not been verified. In this research, we found that unliganded EGFRs may reside outside cholesterol-rich lipid domains of the plasma membranes and can move into lipid raft domains when subjected to ligand binding. Our study provides single-molecule experimental evidence of membrane cholesterol in regulating signaling receptors. Noteworthy, some raftophilic proteins were recently found to appear preferentially at a spatial proximity (<150 nm) to the GM1 nanodomains without physical intermixing [40].

**Fig. 7:**
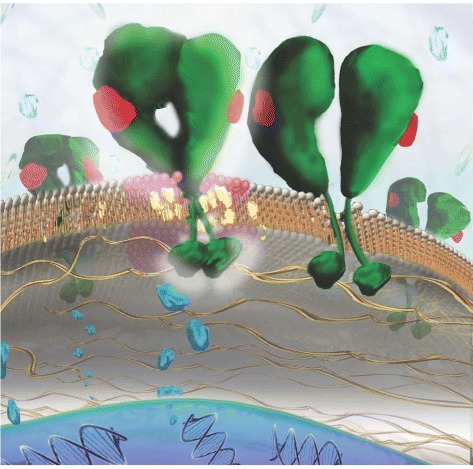
Schematic representation of correlated EGFRs (*green*) in a cholesterol (*yellow*) enriched lipid domain (*center*) and in a cholesterol-depleted nonraft lipid domain (*right*), drawn to illustrate the conclusion of this study. The *red* object on EGFR denotes the EGF ligand. Cholesterol molecules increase receptor-receptor interaction and thus promote the stability of correlatively moving EGFRs

It is appealing to improve our understanding for the mechanism that can drive the ligand bound receptor to lipid domains. Based on the $V(\overline {{{R}_{\tau }}^{2}})$-$\overline {{{R}_{\tau }}^{2}}$ analysis of single-molecule EGFR trajectories in live cells, we proposed a simple molecular-level model. Ligand binding to an EGFR can induce a conformational change of EGFR that may expose some residues with high cholesterol affinity. Thereby, liganded EGFRs may provoke an aggregate of their neighboring raft lipids (such as GM1) to form a cholesterol-rich lipid domain. Figure 7 presents a schematic representation (center) of correlated EGFRs (green) in a cholesterol-rich (yellow) lipid domain. Without membrane cholesterol (right schematic of Fig. 7), a deterministic dimerization interaction dominates, which causes the $V(\overline {{{R}_{\tau }}^{2}})$ value to be increased to higher than 10 as shown in Fig. 6f. Liganded EGFR can also be attracted to an existing lipid domain. As pointed out above that coupling between inner and outer leaves of an asymmetric lipid bilayer could produce inhomogeneities of lipid phases with a nonzero curvature [37]. Such membrane deformations could affect the movements of liganded receptors with the deformed lipids. Because the three cell lines used for this study are quite diverse, our results may be useful to shed light on the mechanism of cholesterol-mediated interaction between activated receptors in live cells.

## Conclusions

In summary, we studied single-molecule optical trajectories of EGFRs moving in the plasma membranes of two cancerous cell lines (A431 and HeLa) and one normal epithelial cell line (MCF-12A). A stochastic model of single-molecule optical data, developed in our previous study [22], was used to analyze and identify critical information from single-molecule trajectories. We disclosed that EGFRs at rest in the three cell lines are located outside the cholesterol-rich lipid domains; EGF binding induces the receptors to move into the cholesterol-rich lipid domains. The liganded receptors diffusing in proximity in the plasma membrane interact with each other that causes the receptors to move correlatively. Membrane cholesterol was found to considerably affect the correlated motion of activated EGFRs. Our single-molecule tracking results reveal the vital role of membrane cholesterol in mediating the interaction between liganded receptors in the three cell lines under study. Because receptor dimerization is a common process for signal transduction, our results can shed light on the way in which cholesterol molecules regulate receptor-receptor interactions in the plasma membranes of live cells.

## Abbreviations

DMEM, Dulbecco’s modified eagle’s medium; EGF, epidermal growth factor; EGFR, epidermal growth factor receptor; EMCCD, electron-multiplying charge-coupled device; M *β*CD, methyl- *β*-cyclodextrin; MD, molecular dynamics; MSD, mean-square displacement; PBS, phosphate buffered saline
